# Varicella zoster virus and influenza vaccine antibody titres in patients from MAGNIFY-MS who were treated with cladribine tablets for highly active relapsing multiple sclerosis

**DOI:** 10.1177/13524585221099413

**Published:** 2022-06-07

**Authors:** Klaus Schmierer, Heinz Wiendl, Celia Oreja-Guevara, Diego Centonze, Anita Chudecka, Sanjeev Roy, Ursula Boschert

**Affiliations:** 1 Centre for Neuroscience, Surgery & Trauma, Blizard Institute, Barts and The London School of Medicine & Dentistry, Queen Mary University of London, London, UK; 2 Clinical Board Medicine (Neuroscience), The Royal London Hospital, Barts Health NHS Trust, London, UK; 3 Department of Neurology, Institute of Translational Neurology, University of Münster, Münster, Germany; 4 Department of Neurology, IdISSC, Hospital Universitario Clinico San Carlos, Madrid, Spain; 5 Departamento de Medicina, Universidad Complutense de Madrid, Madrid, Spain; 6 Laboratory of Synaptic Immunopathology, Department of Systems Medicine, Tor Vergata University, Rome, Italy; 7 Unit of Neurology and Neurorehabilitation, IRCCS Neuromed, Pozzilli, Italy; 8 Clinical Research Services, Cytel Inc., Geneva, Switzerland; 9 Global Medical Affairs, Ares Trading S.A., Eysins, Switzerland (an affiliate of Merck KGaA); 10 Neurology & Immunology, Ares Trading S.A., Eysins, Switzerland (an affiliate of Merck KGaA)

Dear Editor,

The MAGNIFY-MS clinical trial (NCT03364036) aims to determine the onset of action of cladribine tablets 3.5 mg/kg over 2 years (MAVENCLAD®; Merck Europe B.V., Amsterdam, The Netherlands) in patients with highly active relapsing multiple sclerosis. Some patients enrolled in MAGNIFY-MS received vaccinations against varicella zoster virus (VZV) and seasonal influenza as part of their standard of care during the trial, presenting an opportunity to investigate vaccine responses during treatment with cladribine tablets.

Quantitative antibody titre responses to VZV and seasonal influenza vaccines were measured by enzyme-linked immunosorbent and haemagglutination inhibition assays (HAI), respectively. We also explored if the serological response was impacted by lymphocyte counts measured at the time of, or after, vaccination. Ethical approval for MAGNIFY-MS was obtained at each study centre, and all participating patients provided written informed consent.

Blood samples from 14 patients were retrospectively analysed. Three patients received a VZV vaccine (one patient received two doses of the inactivated Shingrix® vaccine; two patients received one dose of the live attenuated Zostavax® vaccine) before initiating treatment with cladribine tablets. All patients mounted seroprotective titres to VZV; the post-vaccination antibody titres of the patient who received Shingrix® were increased > 40-fold over the protective titre at all time points. Seroprotective VZV titres were maintained over the observed post-initiation period with cladribine tablets, despite a marked reduction in lymphocyte counts.

Twelve patients received a seasonal influenza vaccine (one patient received both the VZV and seasonal influenza vaccines). The majority (11/12) had seroprotective antibody titres even before vaccination; post-vaccination seroprotective titres were maintained in those patients. Many patients achieved seroprotection in a short timeframe, that is, between day 21 and 69 from first vaccination. Nine out of 11 patients exhibited a ⩾ twofold titre increase and 4 out of 11 patients exhibited a ⩾ fourfold increase for at least one strain of influenza.

We further observed that seroprotection (or an increase in HAI titres) occurred in both patients who were vaccinated early, that is, up to 6 months after a course of cladribine tablets in Years 1 and 2, and late (months 8.5–10.5 of Year 1). Lymphocyte counts in patients vaccinated late after cladribine tablets were within the normal range at that time. In contrast, patients vaccinated early within the first 6 months after a course of cladribine tablets typically showed grade 1 or 2 lymphopenia. Nevertheless, all patients maintained seroprotection.

Our observations are consistent with recent reports of effective COVID-19 immunisation in patients receiving cladribine tablets.^1,2^ We hypothesise that unique lymphocyte repopulation kinetics induced by cladribine tablets, including incomplete reduction and subsequent rapid recovery of immature B cells,^3^ may explain why vaccination responses appear to resemble those in the normal population while humoral responses in patients treated with other disease-modifying therapies, such as fingolimod and ocrelizumab, with different mechanisms of action, are blunted.^1,2,4,5^

In summary, while results are from a small number of patients vaccinated against VZV and seasonal influenza during treatment with cladribine tablets, they demonstrate consistent humoral responses regardless of timing after treatment administration or total lymphocyte count.

*Full results of this analysis can be found in the Supplementary Material.*

## Supplemental Material

sj-docx-1-msj-10.1177_13524585221099413 – Supplemental material for Varicella zoster virus and influenza vaccine antibody titres in patients from MAGNIFY-MS who were treated with cladribine tablets for highly active relapsing multiple sclerosis Click here for additional data file. Supplemental material, sj-docx-1-msj-10.1177_13524585221099413 for Varicella zoster virus and influenza vaccine antibody titres in patients from MAGNIFY-MS who were treated with cladribine tablets for highly active relapsing multiple sclerosis by Klaus Schmierer, Heinz Wiendl, Celia Oreja-Guevara, Diego Centonze, Anita Chudecka, Sanjeev Roy and Ursula Boschert in Multiple Sclerosis Journal
