# Supplementary material for: Varicella zoster virus and influenza vaccine antibody titres in patients from MAGNIFY-MS who were treated with cladribine tablets for highly active relapsing multiple sclerosis
Source: Mult Scler. 2022 Jun 7;28(13):2151–3. doi: 10.1177/13524585221099413 (PMC9574232; doi:10.1177/13524585221099413)
Supplement: sj-docx-1-msj-10.1177_13524585221099413 – Supplemental material for Varicella zoster virus and influenza vaccine antibody titres in patients from MAGNIFY-MS who were treated with cladribine tablets for highly active relapsing multiple sclerosis [file sj-docx-1-msj-10.1177_13524585221099413.docx]

Supplementary Material

Varicella Zoster Virus and Influenza Vaccine Antibody Titres in Patients From MAGNIFY-MS who Were Treated With Cladribine Tablets for Highly Active Relapsing Multiple Sclerosis

It should be noted that presented data are limited to the small number of patients from the study and that not all patients had received the full 2 years of treatment with cladribine tablets, as the trial was still ongoing at the time of analysis.

Supplementary Figure 1. Administration and Sampling Timeline for Vaccinations

#
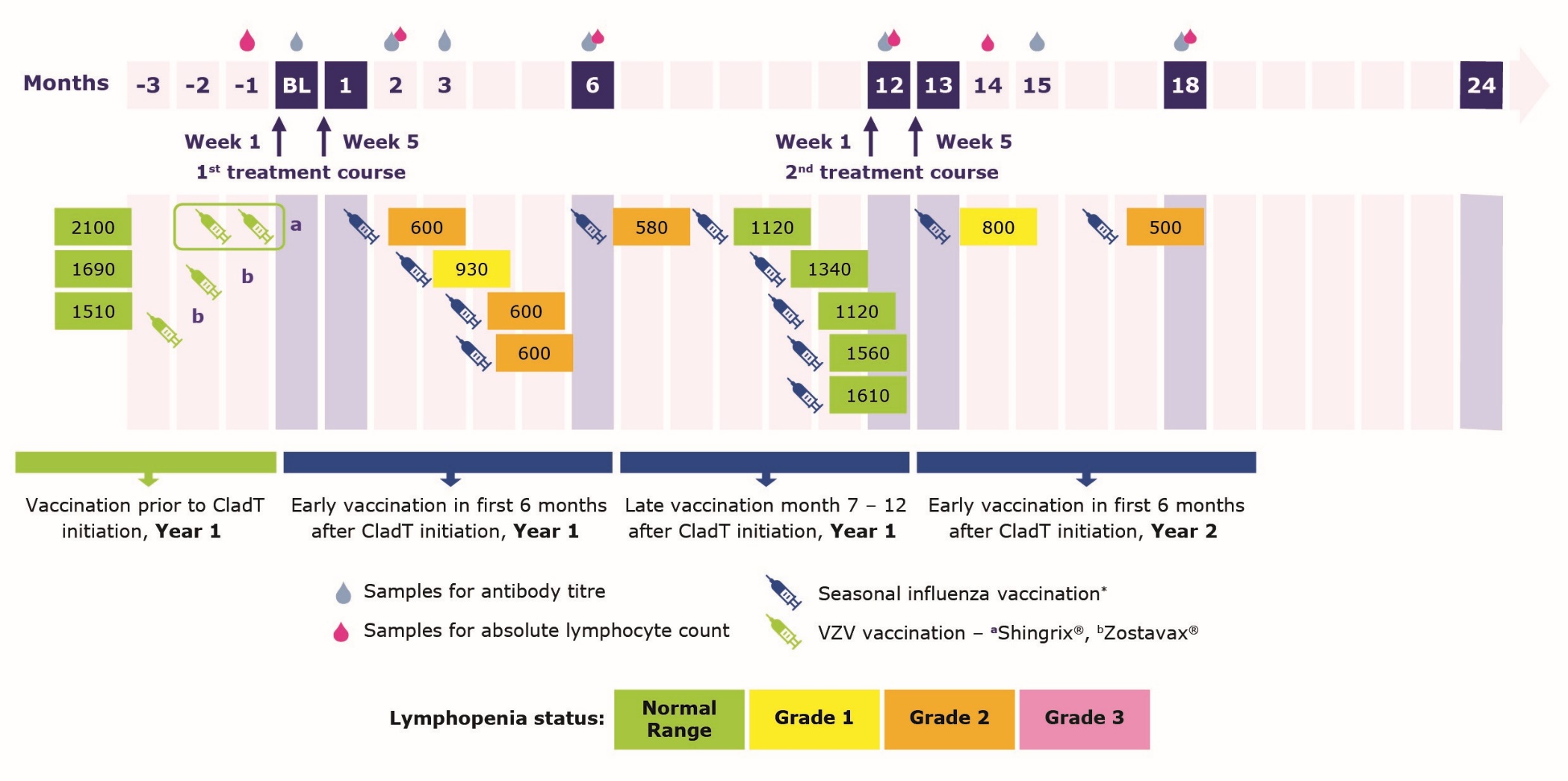


Each syringe represents a different patient (with the exception of the Shingrix® vaccine). Needle tips indicate timing of vaccination. Absolute lymphocyte counts (cells/μL) are represented next to each syringe.

*The vaccine strains for seasonal influenza differed each year.

Lymphopenia: grade 1, 800–999 cells/μL; grade 2, 500–799 cells/μL; grade 3, 200–499 cells/μL; and grade 4, <200 cells/μL.

BL, baseline; CladT, cladribine tablets; VZV, varicella zoster virus

**Supplementary Figure 2. VZV Vaccination Before Initiation of Year 1 of Treatment With Cladribine Tablets**


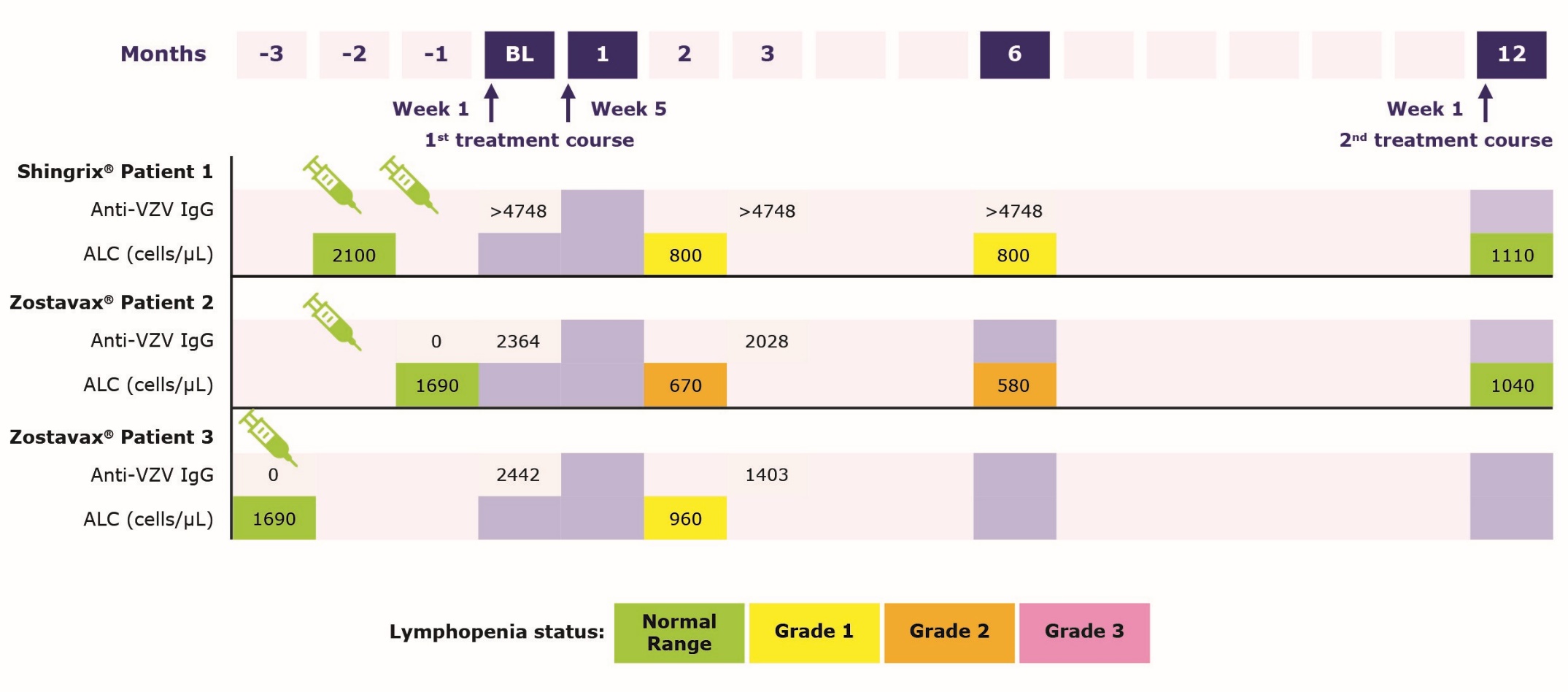


Needle tips indicate timing of vaccination.

Lymphopenia: grade 1, 800–999 cells/μL; grade 2, 500–799 cells/μL; grade 3, 200–499 cells/μL; and grade 4, <200 cells/μL.

ALC, absolute lymphocyte count; BL, baseline; IgG, immunoglobulin G; VZV, varicella zoster virus

**Supplementary Figure 3. ALC, B cell, and T cell Counts at Seasonal Influenza Vaccination During or After Cladribine Tablets Treatment Course in Year 1 and Year 2**


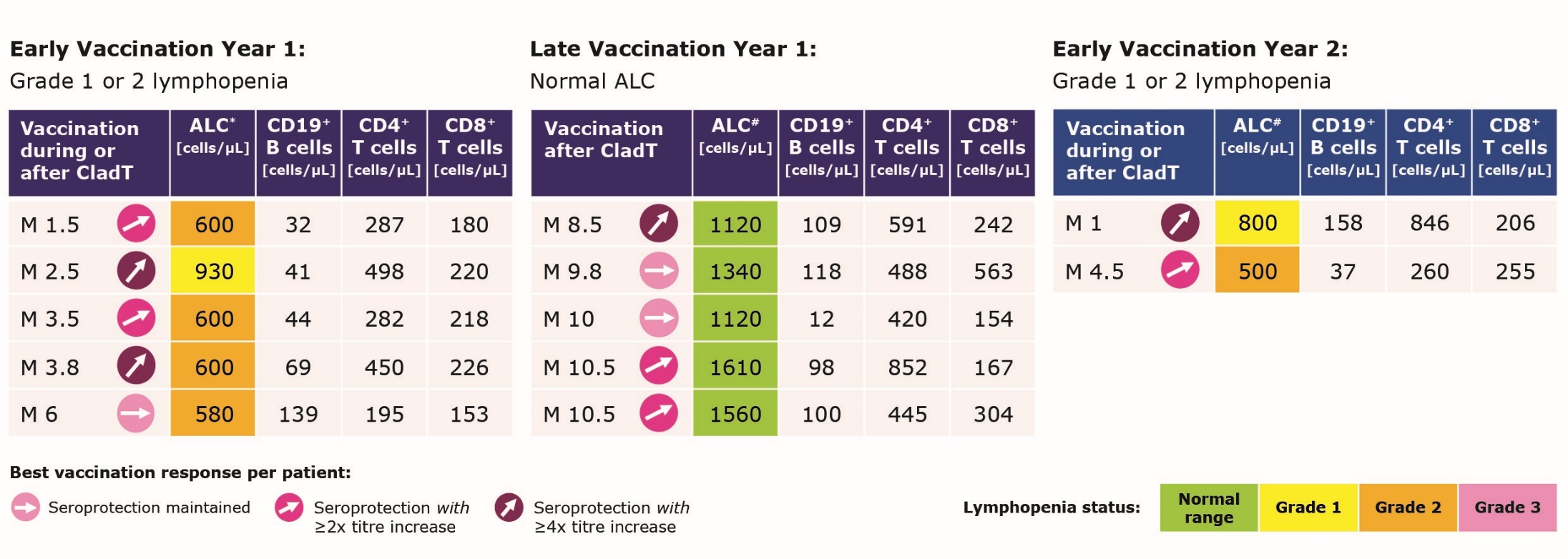


Approx. ALC defined with pre- and/or post-vaccination time points: *ALC up to +/- 0.5 M from vaccination; ^#^ALC up to +/- 2 M from vaccination.

Lymphopenia: grade 1, 800–999 cells/μL; grade 2, 500–799 cells/μL; grade 3, 200–499 cells/μL; and grade 4, <200 cells/μL. Normal limits for other cells: CD19^+^ B cells, 80–616 cells/μL; CD4^+^ T cells, 404–1612 cells/μL; and CD8^+^ T cells, 220–1129 cells/μL.

ALC, absolute lymphocyte count; CladT, cladribine tablets; M, month

**Supplementary Table 1. Humoral immune response to seasonal influenza vaccination: Seroprotection rate**

| **Parameter** | **Last Pre-Vaccination Sample^*^** | | **Post-Vaccination Samples** | | | |
| --- | --- | --- | --- | --- | --- | --- |
|  |  |  | **First Post-Vaccination Sample^†^** | | **Alternative Post-Vaccination Sample^‡^** | |
|  | **n/N (%)** | **95% Confidence Interval** | **n/N (%)** | **95% Confidence Interval** | **n/N (%)** | **95% Confidence Interval** |
| Seroprotection rate in at least one strain | 11/11 (100) | [71.5, 100] | 12/12 (100) | [73.5, 100] | 10/10 (100) | [69.2, 100] |
| Seroprotection rate in at least one HAI A strain | 11/11 (100) | [71.5, 100] | 12/12 (100) | [73.5, 100] | 10/10 (100) | [69.2, 100] |
| Seroprotection rate in at least one HAI B strain | 10/11 (90.9) | [58.7, 99.8] | 11/12 (91.7) | [61.5, 99.8] | 9/10 (90.0) | [55.5, 99.7] |

As per the EMA cut-off, the seroprotection titre level is considered ≥40 for the seasonal influenza vaccine.

^*^Last pre-vaccination sample: last non-missing value of antibody titre before first vaccination date and after the baseline. ^†^First post-vaccination sample: first non-missing value of antibody titre after first vaccination date (between 21 and 198 days [~0.7 – 6.6 months] from first vaccination). ^‡^Alternative post-vaccination sample: second next non-missing value of antibody titre after first vaccination date (between 111 and 289 days [~3.7 – 9.6 months] from first vaccination).

EMA, European Medicines Agency; HAI, haemagglutination inhibition assay

**Supplementary Table 2. Humoral immune response to seasonal influenza vaccination**

| **Parameter** | **Last pre-vaccination sample^*^ vs. First post-vaccination sample**^†^ | | **Last pre-vaccination sample vs. Alternative post-vaccination sample**^‡^ | |
| --- | --- | --- | --- | --- |
|  | **n/N (%)** | **95% Confidence**  **Interval** | **n/N (%)** | **95% Confidence**  **Interval** |
| Patients who seroconverted with a ≥4-fold increase in serum antibody titre in ≥1 strain | 4/11 (36.4) | [10.9, 69.2] | 2/ 9 (22.2) | [2.8, 60.0] |
| Patients who seroconverted with a ≥4-fold increase in serum antibody titre in ≥1 HAI A strain | 4/11 (36.4) | [10.9, 69.2] | 2/ 9 (22.2) | [2.8, 60.0] |
| Patients who seroconverted with a ≥4-fold increase in serum antibody titre in ≥1 HAI B strain | 1/11 (9.1) | [0.2, 41.3] | 0/ 9 (0) | - |
| **Sensitivity Analysis** | | | | |
| Patients with a ≥2-fold increase in serum antibody titre in ≥1 strain | 9/11 (81.8) | [48.2, 97.7] | 4/ 9 (44.4) | [13.7, 78.8] |
| Patients with a ≥2-fold increase in serum antibody titre in ≥1 HAI A strain | 8/11 (72.7) | [39.0, 94.0] | 4/ 9 (44.4) | [13.7, 78.8] |
| Patients with a ≥2-fold increase in serum antibody titre in ≥1 HAI B strain | 4/11 (36.4) | [10.9, 69.2] | 3/ 9 (33.3) | [7.5, 70.1] |

^*^Last pre-vaccination sample: last non-missing value of antibody titre before first vaccination date and after the baseline. ^†^First post-vaccination sample: first non-missing value of antibody titre after first vaccination date. ^‡^Alternative post-vaccination sample: second next non-missing value of antibody titre after first vaccination date.

HAI, haemagglutination inhibition assay

**Supplementary Table 3. Anti-VZV titres and immune cell counts for an example patient who received the Shingrix® vaccine before treatment with cladribine tablets**Patient was vaccinated 68 and 8 days before the first dose of cladribine tablets (course 1)

| **Trial month** | **Scr** | **BL** | **1** | **2** | **3** | **6** | **12** | **14** | **15** | **18** | **End** |
| --- | --- | --- | --- | --- | --- | --- | --- | --- | --- | --- | --- |
| **Anti-VZV IgG [IU/L]** |  | **>4748** |  |  | **>4748** | **>4748** |  |  |  |  |  |
| **IgG [g/L]** |  | 14.1 |  |  | 15.1 | 14.6 |  |  | 14.3 | 14.1 |  |
| **ALC [cells/µL]** | 2100 |  |  | 800 |  | 800 | 1110 | 300 |  | 700 |  |
| **CD19^+^ B cells [cells/µL]** |  | 504* |  |  | 39 | 97 |  |  | 80 | 215 |  |
| **Memory B cells [cells/µL]** |  | 66.99672 |  |  | 1.94142 | 1.83815 |  |  | 1.32880 | 2.99065 |  |
| **Naïve B cells [cells/µL]** |  | 160.65504 |  |  | 35.23455 | 90.12755 |  |  | 77.77360 | 209.63360 |  |
| **CD69^+^ activated B cells [cells/µL]** |  | 2.97864 |  |  | 0.2574 | 11.57986 |  |  | 1.3808 | 5.6373 |  |
| **PCs (CD20^-^CD38^bright^) [cells/µL]** |  | 230.25744 |  |  | 0.28587 | 0.24638 |  |  | 0.26400 | 0.42355 |  |
| **Short-lived PCs (CD20^-/dim^CD27^bright^) [cells/µL]** |  | 251.97984 |  |  | <LLOQ | 0.22310 |  |  | <LLOQ | 0.46870 |  |
| **CD16^+^ CD56^+^ NK cells [cells/µL]** |  | 243 |  |  | 107 | 145 |  |  | 146 | 90 |  |
| **CD4^+^ T cells [cells/µL]** |  | 956 |  |  | 274 | 249 |  |  | 164 | 175 |  |
| **CD8^+^ T cells [cells/µL]** |  | 655 |  |  | 241 | 251 |  |  | 179 | 169 |  |

**Supplementary Table 3. Contd**

VZV titres shown in **bold** are post-vaccination.

BL blood was collected up to 6 days before first cladribine tablets dose. Date of ALC measurements may vary from other measurements by up to 7 days.

^*^CD19^+^ B cells come from a TBNK panel in comparison to other biomarkers (i.e. B cell panel).

ALC, absolute lymphocyte count; BL, baseline; End, end of trial; IgG, immunoglobulin G; LLOQ, lower limit of quantitation; NK, natural killer; PC, plasma cell; Scr, screening; VZV, varicella zoster virus

**Supplementary Table 4. HAI titres and immune cell counts for an example patient who received the seasonal influenza vaccine during Year 1 of treatment with cladribine tablets**Patient was vaccinated 72 days after the first dose of cladribine tablets (course 1) against influenza strains H1N1 (A/Michigan), H3N2 (A/Singapore), Victoria (B/Colorado), and Yamagata (B/Phuket)

| **Trial month** | **Scr** | **BL** | **1** | **2** | **3** | **6** | | **12** | | **14** | | **15** | | **18** | | **End** | |  |
| --- | --- | --- | --- | --- | --- | --- | --- | --- | --- | --- | --- | --- | --- | --- | --- | --- | --- | --- |
| **H1N1 (A/Michigan)** |  | 240 |  | 80 | **>1280** | | **>1280** | |  | |  | |  | |  | |  | |
| **H3N2 (A/Singapore)** |  | 40 |  | 20 | **80** | | **40** | |  | |  | |  | |  | |  | |
| **Victoria (B/Colorado)** |  | 20 |  | 20 | **40** | | **60** | |  | |  | |  | |  | |  | |
| **Yamagata (B/Phuket)** |  | 20 |  | 30 | **40** | | **40** | |  | |  | |  | |  | |  | |
| **IgG [g/L]** |  | 11 |  |  | 10.8 |  | | 10.8 | | 10.5 | | 12.4 | | 10.8 | | 10.5 | |  |
| **ALC [cells/µL]** | 1420 |  |  | 930 |  | 800 | | 900 | | 750 | |  | | 710 | | 740 | |  |
| **CD19^+^ B cells [cells/µL]** |  | 377* | 135 | 41 | 40 | 64 | | 134 | | 25 | | 28 | | 59 | | 79 | |  |
| **Memory B cells [cells/µL]** |  | 116.45907 | 40.67955 | 11.95314 | 8.40920 | 8.64640 | | 19.46082 | | 6.79225 | | 5.11000 | | 8.69070 | | 12.39905 | |  |
| **Naïve B cells [cells/µL]** |  | 243.64002 | 82.25550 | 23.64306 | 26.90360 | 49.16608 | | 108.54938 | | 14.93125 | | 20.38092 | | 45.68606 | | 59.16310 | |  |
| **CD69^+^ activated B cells [cells/µL]** |  | 5.31570 | 2.45970 | 0.99097 | 1.00360 | 0.79232 | | 2.83678 | | 0.61350 | | 0.64036 | | 0.91214 | | 1.42674 | |  |
| **PCs (CD20^-^CD38^bright^) [cells/µL]** |  | 3.18565 | 2.87550 | 1.05739 | 0.74480 | 2.96320 | | 0.66062 | | 1.05400 | | 0.38472 | | 0.67024 | | 2.27441 | |  |

**Supplementary Table 4. Contd**

| **Trial month** | | **Scr** | | **BL** | | **1** | | **2** | | **3** | | **6** | | **12** | | **14** | | **15** | | **18** | | **End** | |
| --- | --- | --- | --- | --- | --- | --- | --- | --- | --- | --- | --- | --- | --- | --- | --- | --- | --- | --- | --- | --- | --- | --- | --- |
| **Short-lived PCs (CD20^-/dim^CD27^bright^) [cells/µL]** |  | | 2.95945 | | 2.99430 | | 0.95981 | | 0.58480 | | 0.175744 | | 0.87234 | | 0.98750 | | 0.33684 | | 0.37406 | | 1.97184 | |  |
| **CD16^+^ CD56^+^ NK cells [cells/µL]** |  | | 234 | | 146 | | 100 | | 75 | | 136 | | 130 | | 162 | | 96 | | 159 | | 171 | |  |
| **CD4^+^ T cells [cells/µL]** |  | | 992 | | 682 | | 498 | | 268 | | 323 | | 411 | | 376 | | 338 | | 353 | | 362 | |  |
| **CD8^+^ T cells [cells/µL]** |  | | 477 | | 319 | | 220 | | 123 | | 159 | | 173 | | 182 | | 193 | | 176 | | 173 | |  |

HAI titres shown in **bold** are post-vaccination.

BL blood was collected up to 6 days before first cladribine tablets dose. Date of ALC measurements may vary from other measurements by up to 7 days.

^*^CD19^+^ B cells come from a TBNK panel in comparison to other biomarkers (i.e. B cell panel).

ALC, absolute lymphocyte count; BL, baseline; End, end of trial; HAI, haemagglutination inhibition; IgG, immunoglobulin G; NK, natural killer; PC, plasma cell; Scr, screening

**Supplementary Table 5. HAI titres and immune cell counts for an example patient who received the seasonal influenza vaccine at Year 2 of cladribine tablets treatment**

Patient was vaccinated 33 days after first dose of cladribine tablets (course 2) against influenza strains H1N1 (A/Brisbane), H3N2 (A/Kansas), Victoria (B/Colorado), and Yamagata (B/Phuket)

| **Trial month** | **Scr** | **BL** | **1** | **2** | **3** | **6** | **12** | **14** | **15** | **18** | **End** |
| --- | --- | --- | --- | --- | --- | --- | --- | --- | --- | --- | --- |
| **H1N1 (A/Brisbane)** |  | 160 |  |  |  |  | >1280 |  | **>1280** | **>1280** |  |
| **H3N2 (A/Kansas)** |  | 80 |  |  |  |  | 80 |  | **640** | **320** |  |
| **Victoria (B/Colorado)** |  | 40 |  |  |  |  | 40 |  | **40** | **80** |  |
| **Yamagata (B/Phuket)** |  | 40 |  |  |  |  | 80 |  | **80** | **80** |  |
| **IgG [g/L]** |  | 8.3 |  |  | 10.6 | 10.3 | 10.3 |  | 10.6 | 11.1 |  |
| **ALC [cells/µL]** | 2120 |  |  | 1290 |  | 1170 | 1280 | 790 |  | 1010 | 1560 |
| **CD19^+^ B cells [cells/µL]** |  | 415* |  |  |  | 67 | 158 |  |  | 152 |  |
| **Memory B cells [cells/µL]** |  | 103.39310 |  |  |  | 5.18245 | 7.75622 |  |  | 3.32424 |  |
| **Naïve B cells [cells/µL]** |  | 278.77625 |  |  |  | 53.37488 | 143.27440 |  |  | 141.43448 |  |
| **CD69^+^ activated  B cells [cells/µL]** |  | 4.75175 |  |  |  | 1.12694 | 2.24834 |  |  | 1.87112 |  |
| **PCs (CD20^-^CD38^bright^) [cells/µL]** |  | 5.81415 |  |  |  | 1.84250 | 0.81686 |  |  | 1.26008 |  |
| **Short-lived PCs (CD20^-/dim^CD27^bright^) [cells/µL]** |  | 2.80540 |  |  |  | 1.90816 | 0.91324 |  |  | 0.96672 |  |
| **CD16^+^ CD56^+^ NK cells [cells/µL]** |  | 174 |  |  |  | 63 | 75 |  |  | 136 |  |

**Supplementary Table 5. Contd**

| **Trial month** | **Scr** | | **BL** | | **1** | | **2** | | **3** | | | **6** | **12** | | | **14** | | **15** | | **18** | **End** | |  |
| --- | --- | --- | --- | --- | --- | --- | --- | --- | --- | --- | --- | --- | --- | --- | --- | --- | --- | --- | --- | --- | --- | --- | --- |
| **CD4^+^ T cells [cells/µL]** | |  | | 1295 | |  | |  | |  | 693 | | | 846 |  | |  | | 559 | | |  | |
| **CD8^+^ T cells [cells/µL]** | |  | | 414 | |  | |  | |  | 194 | | | 206 |  | |  | | 129 | | |  | |

HAI titres shown in **bold** are post-vaccination.

BL blood was collected up to 6 days before first cladribine tablets dose. Date of ALC measurements may vary from other measurements by up to 7 days.

^*^CD19^+^ B cells come from a TBNK panel in comparison to other biomarkers (i.e. B cell panel).

ALC, absolute lymphocyte count; BL, baseline; End, end of trial; HAI, haemagglutination inhibition; IgG, immunoglobulin G; NK, natural killer; PC, plasma cell; Scr, screening
